# CT assessment of the increased density of cerebral vessels in plateau region

**DOI:** 10.1038/s41598-021-85448-3

**Published:** 2021-03-12

**Authors:** Haiting Zhou, Tsering Tashi, Deli Zhao, Sonam Tsring, Hongwei Liang, Jinling Zhang

**Affiliations:** 1 Department of Computed Tomography, The Second Affiliated Hospital of Harbin Medical University, Harbin, 150086 China; 2 Department of Radiology, Rinbung County Health Service Center, Xigaze, Tibet, 857200 China

**Keywords:** Neurology, Diseases, Neurological disorders

## Abstract

In this study, the relationship between the brain parenchymal density, the cerebral vessel density, the mean corpuscular hemoglobin (MCH) content, the mean corpuscular hemoglobin concentration (MCHC), and the morbidity associated with lacunar infarction of residents living in either the plains or the plateau regions were analyzed and compared for their potential clinical implications. Clinical data from the brain CT scans of individuals living in either the plain or plateau regions (129 each) were collected. Specifically, the CT values for basal ganglia, the middle cerebral artery, and the superior sagittal sinus, along with the number of patients with lacunar infarction, were collected. In addition, the MCH and MCHC values were measured in blood samples collected within 48 h following the CT scans. For statistical analysis, an independent sample t-test, Pearson's correlation test (permutation test), and Chi-squared test were employed. The inhabitants of the plateau had a significantly higher CT value of basal ganglia, the middle cerebral artery, and superior sagittal sinus and also higher levels of MCH and MCHC in the blood (*ps* < 0.001) than the inhabitants of the plains region. Further, there was a significant positive correlation between the three aforementioned CT values and the MCH and MCHC findings. However, no significant differences were found in the morbidity of lacunar infarction between these two regions (*p* > 0.05). The inhabitants in the plateau have a significantly higher brain parenchymal density, higher CT value for cerebral vessels density, and higher blood MCH and MCHC levels in comparison with individuals occupying the plains. Concurrently, the parenchymal density and the CT values are shown to be positively correlated with the MCH and MCHC content in the blood.

## Introduction

Thanks to a surge in economic development and increased availability of medical imaging resources in the plateau regions of China, brain CT scans have become one of the standard medical imaging modalities in this region. However, one of the common contentions associated with the widespread use of brain CT scans is that the relatively high-density blood vessel in the brain tissue can be misinterpreted or misdiagnosed as either pathological high-density blood vessels or brain sulcus. Further compounding the problem is that the morbidity of lacunar infarction in the plateau regions is also relatively high^1^. As such, it is imperative that accurate diagnosis is rendered. Therefore, this study was carried out to differentiate the diagnosis between the physiological and pathological features of blood vessels in the brain and their underlying reasons in CT scans in order to help establish a robust system to prevent excessive medical care and misdiagnosis.

## Materials and methods

The medical data for 129 residents living in the plains region, with an altitude ranging from 132 to 500 m, were collected between the months of August and November in 2018, of which 59 participants were males and 70 were females. Forty participants were placed in the young group (18 to 44 years), thirty-two participants were in the middle-age group (45 to 59 years), and fifty-seven participants were in the senior-age group (60 years and older). In parallel, the medical data of 129 inhabitants of the plateau region, with an altitude ranging from 3761 to 4200 m, were collected between June and September in 2018, in which there were 95 men and 34 women. Of these, forty participants were placed in the young group, thirty-two participants were in the middle-age group, and fifty-seven participants were in the senior-age group. The participants included in this study were all randomly included. Due to the limited number of patients in plateau area, in order to ensure the sample size, as many patients as possible were selected into the group, so they were randomly included. In order to keep the sample of plain area close to plateau area and reduce human variables, plain area was also randomly selected.In this manuscript, the data of plateau group were collected by the first author and colleagues of corresponding hospital during the period of supporting Tibet, while the data of plain group were collected by corresponding author and colleagues in this unit. This study was approved by the Ethics Committee of The Second Affiliated Hospital of Harbin Medical University and Rinbung County Health Service Center. All experiments were performed in accordance with relevant guidelines and regulations and all patients were required to sign informed consent forms.

### Image acquisition

The participants in the plain region were scanned using a 16-Slice CT from GE with 120 kV, 280 mA, 5 mm slice thickness, and 5 mm slice interval, while the participants in the plateau were scanned by Somatom Scope, a 16-slice CT from Siemens, with 130 kV, 100–120 mA, 4.8 mm slice thickness, and 4.8 mm slice interval.

For both groups (plain versus plateau), the CT images were interpreted independently by two radiologists, both with at least five years of experience. The blood tests were conducted within the 48 h following the CT scan, and automated hematology analyzers (Sysmex XE-5000 for plain versus Dirui BF-6700 for plateau) were used to obtain the MCH and MCHC values.

### CT image analysis

**Figure 1:**
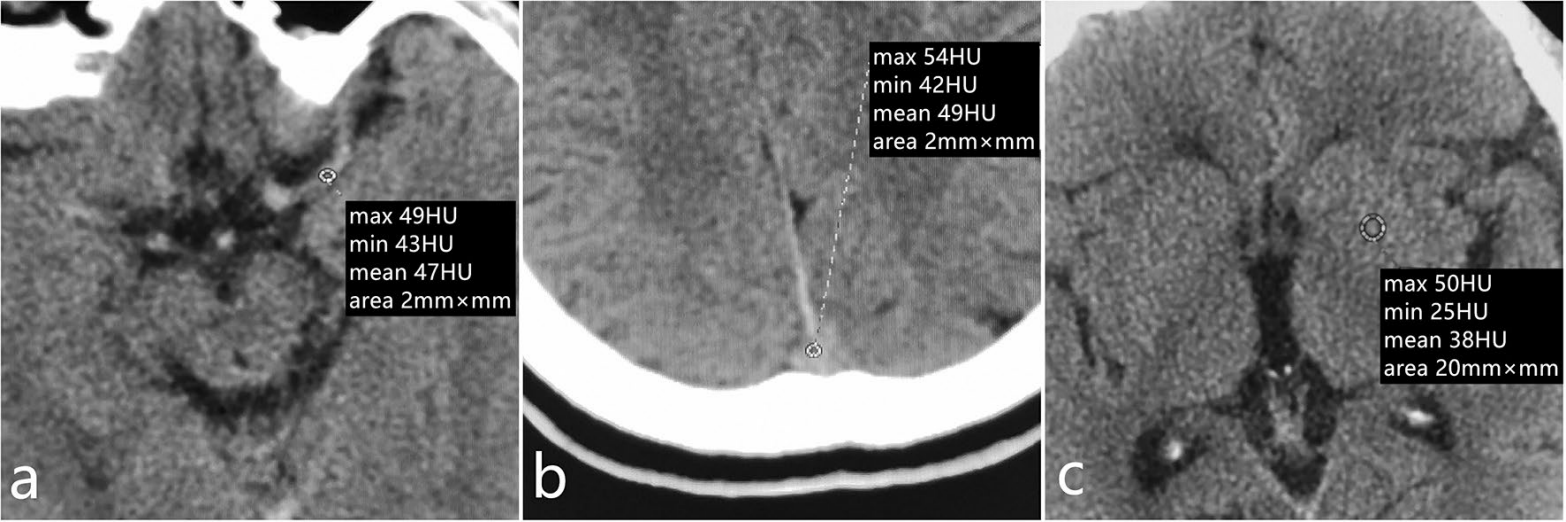
The CT values for basal ganglia, the middle cerebral artery, and the superior sagittal sinus were collected. The middle cerebral artery (**a**), the superior sagittal sinus (**b**), basal ganglia (**c**).

The CT values for basal ganglia, the middle cerebral artery, and the superior sagittal sinus were collected. Three CT measurements were performed on one side of the visual symmetry of the regions of interest in both cerebral hemispheres. For the middle cerebral artery and superior sagittal sinus, the scan area was 2 mm^2^; for the basal ganglia, the scan area was 20 mm^2^. Every scan was repeated 3 times to acquire the mean value corresponding to a particular region (Fig. 1).Superior The middle cerebral artery (MCA) is separated from the internal carotid artery and runs in the lateral fissure of the brain. The M1 segment of MCA runs horizontally on the axial image. There are cerebrospinal fluid space and brain parenchyma space around this segment, which is easy to display and measure. Sagittal sinus is one of the thickest venous sinuses in the brain. It contains most of the venous blood in the lateral and medial dorsal sides of the brain. It can reflect the CT value of intracranial venous vessels and is easy to measure. The basal ganglia is the gray matter nucleus in the center of the brain. The density is uniform and the area is large on CT images. It is not easy to have the partial volume effect of gray and white matter mixed together when measuring. Therefore, the above three areas are selected as the measurement area.The participants were screened for lacunar infarction and their respective blood MCH and MCHC values were measured within 48 h of the CT scan.

### Statistical analysis

The SPSS 26.0 statistical software was used in this study for statistical analysis, and all data were presented as means and standard deviation (mean ± SD). All CT values for the basal ganglia, the middle cerebral artery, and superior sagittal sinus, along with blood MCH and MCHC levels from both groups were statistically analyzed via an independent sample t-test for which and an alpha of 0.05 was used as the cut-off for significance. Moreover, Pearson's correlation test was applied to determine the correlation coefficient between the CT values of basal ganglia, the middle cerebral artery, superior sagittal sinus, and the blood MCH and MCHC levels. Finally, a Chi-squared test (*χ*^2^) was used to check if there was a significant difference in the morbidity of lacunar infarction between the inhabitants of the plains versus the plateau.

## Results

**Figure 2:**
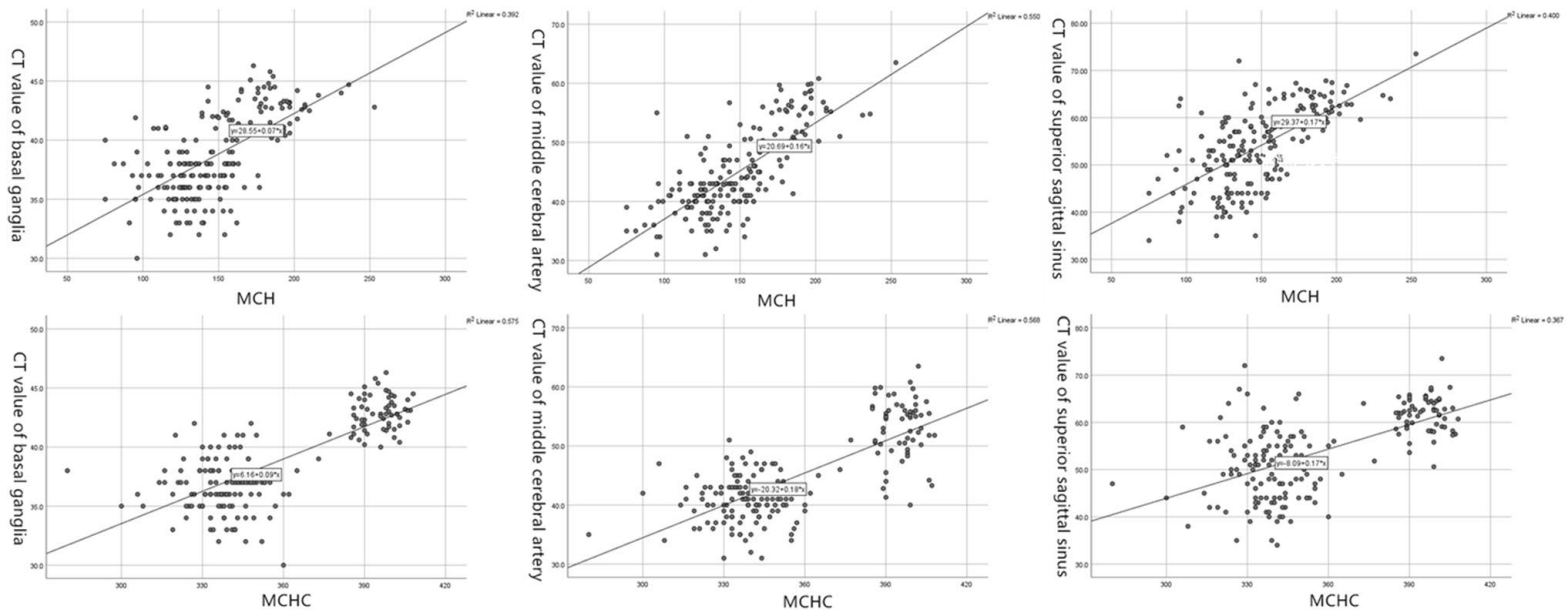
Scatter plot revealed a positive correlation between the CT values of basal ganglia, the middle cerebral artery, and the superior sagittal sinus and MCH and MCHC in all patients of the two groups.

As shown in Table 1and Fig. 2, the CT values of basal ganglia, the middle cerebral artery, and superior sagittal sinus, along with the MCH and MCHC values derived from blood tests were represented as variance all these five parameters were significantly high in the plateau group (*ps* < 0.001). The three CT values for basal ganglia, the middle cerebral artery, and superior sagittal sinus were found to be positively correlated with the two parameters from the blood test, namely, the MCH and MCHC (Table 2). As shown in Table 3, There were 67 cases of lacunar infarction from the plain group (51.9%), while 74 cases were observed in the plateau group (57.4%), and there was no significant difference between these two groups(*χ*^2^(1) = 0.766, *p* > 0.05).

**Table 1:** The statistics of the following five parameters from both plateau and plain groups.

Groups	*N*	Mean value	SD	Mean standard error	*t*
**Basal ganglia**					
Plain	129	36.698	2.2382	0.1971	− 22.823***
Plateau	129	42.336	1.6925	0.1490	
**Middle cerebral artery**					
Plain	129	40.899	3.8096	0.3354	− 13.263***
Plateau	129	50.088	6.8852	0.6062	
**Superior sagittal sinus**					
Plain	129	50.0388	7.26873	0.63998	− 13.946***
Plateau	129	60.6822	4.72315	0.41585	
**MCH**					
Plain	129	131.95	20.768	1.829	− 13.828***
Plateau	61	180.59	26.185	3.353	
**MCHC**					
Plain	129	337.961	12.9527	1.1404	− 11.855***
Plateau	61	389.651	45.9273	5.8804

**Table 2:** The correlation matrix of CT values from basal ganglia, middle cerebral artery, and superior sagittal sinus, and the MCH and MCHC values from the blood test.

	Basal ganglia	Middle cerebral artery	Superior sagittal sinus
**MCH**			
Pearson Correlation	.626***	.742***	.632***
N	190	190	190
**MCHC**			
Pearson Correlation	.758***	.754***	.606***
* N*	189	189	189

(*p < 0.05, **p < 0.01, and ***p < 0.001).

**Table 3:** The list of lacunar infarction against groups.

	Lacunar infarction		
	No	Yes	Total
**Groups**			
Plain			
Frequency	62	67	129
%	48.1%	51.9%	100.0%
Plateau			
Frequency	55	74	129
%	42.6%	57.4%	100.0%
**Total**			
Frequency	117	141	258
%	45.3%	54.7%	100.0%

## Discussion

**Figure 3:**
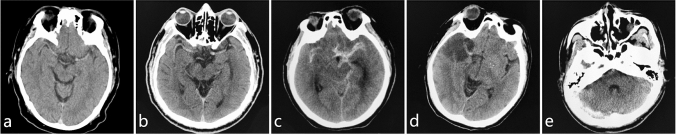
The participants in the plateau group had a higher density of cerebral vessels (**a**). The high brain blood vessel density was still apparent due to the residual contrast agent (**b**). Subarachnoid haemorrhage (**c**). The hyperdense artery sign is the feature of the infarcted blood vessel, while there should be ischemic features at the aerenchyma that the blood vessel irrigates (**d**). Transverse sinus in the right side sinus thrombosis (**e**).

It is a common phenomenon that long-term inhabitants of plateaus tend to manifest an increased density of blood vessels in the brain. In particular, we observed that the young participants in the plateau group (18–44 years of age) had a higher density of cerebral vessels, with no discernable signs of cerebral infarction, cerebral hemorrhage, cerebral atrophy in the medical imaging (Fig. 3a). Thus, the normal physiologic increase of cerebrovascular density should be distinguished from the residual of contrast agent, subarachnoid hemorrhage, hyperdense artery sign, or cerebral venous sinus thrombosis. Specifically, for the residual contrast agent used in the CT scan. When the brain parenchymal density declined to levels comparable to those in plain CT values, the high brain blood vessel density was still apparent due to the residual contrast agent (Fig. 3b). In cases presenting with subarachnoid hemorrhage,. Since the large blood vessels are under sulcus and cranial plate, it may be an encumbrance to clearly distinguish the high density of blood vessels from subarachnoid hemorrhage when the sulcus are not wide enough, and the density of sulcus is high. Further, the hyperdense artery sign is always attributed to the arterial thrombus and leads to cerebral infarction^2–4^. Apart from the arterial thrombus, the morbidity age of cerebral venous sinus thrombosis, a hemostatic abnormality, was found to be relatively young and appears to be more common in women. The blood vessel density is higher around the thrombus. However, since the blood vessel density in inhabitants of the plateaus is commonly higher than normal for both veins and arteries, this needs to be distinguished from the aforementioned pathological signs.

An increase in altitude is associated with lowered atmospheric pressure, reduced oxygen availability in the air, and decreased ambient temperature^5^. The resulting physiological adaptations to these conditions is termed acclimatization^6–8^. Conversely, people may lose acclimatization after living at high altitudes for prolonged periods, with symptoms such as erythrocytosis, headache, dizziness, insomnia, pulmonary arterial hypertension, and severe hypoxemia. These symptoms can be diagnosed as chronic mountain sickness (CMS), as per the Qinghai Chronic Mountain Sickness Scoring System^9, 10^.

This study demonstrated that MCH for inhabitants living in the plateau is significantly higher than that of the inhabitants in the plain region, which may be attributed to the acclimatization to hypoxia and the CMS. It is known that the brain blood vessel density is determined by the CT value of blood and the density of a liquid is determined by its constituents. Blood mainly consists of erythrocytes and plasma, and the latter is mostly composed of water, for which the CT value is 0 HU. In conclusion, the primary determinant of blood density is the concentration of hemoglobin^11, 12^. This study demonstrated a positive correlation between blood vessel density and hemoglobin concentration. Hence, the life-history of the patients and their blood test results should be considered. Further, the recent administration of the CT contrast agent should be taken into consideration. On the other side, in patients with subarachnoid hemorrhage, the increased density of sulcus is limited and restricted to the vicinity of the lesion, while the edge of the hematocele with high-density appears less sharp than the common high density of blood vessel (Fig. 3c). In addition, the hyperdense artery sign or venous sinus thrombosis is almost always very limited, which is easy to be distinguished by the overall increased blood vessel density (Fig. 3d,e). Typically, the hyperdense artery sign is the feature of the infarcted blood vessel, while there should be ischemic features at the parenchyma that the blood vessel irrigates.

Cranial nerve nucleus is the essential functional area in the parenchyma. Due to its high density of cerebral vessels and priority for blood supply during the anaerobic exercise, the density of the basal ganglia of residents living in the plateau is significantly higher than those occupying the plains^13, 14^.

Due to its high metabolic activity and heavy dependence on oxygen, the brain has increased blood viscosity and there is a risk of vasospasm and thrombus for inhabitants living in areas with low oxygen^15^, which likely leads to the high morbidity of infarction observed in the plateau regions. However, in this study, there was no significant difference in the morbidity of infarction. The increased morbidity associated with arteriosclerosis and infarction in a specific plain region may be attributed to increased temperature differences between winter and summer months and between household and external weather conditions during winter, as well as the high-salt diet, all of which may obscure or mask any potential effects caused by living in different altitudes.

Different models of CT scanners were used to collect the data in this study due to the restrictions induced by various terrains, but all CT scanners had been calibrated beforehand, which should have a non-significant effect on the CT value measurements. The data for the plain group was collected from Northeast China, where the climate and the habitat may affect the morbidity of arteriosclerosis and infarction. Further, the data size for blood tests corresponding to the plateau group is slightly smaller than the one from the plain region, which, however, is not a limitation for purposes of statistical analysis.

## Conclusion

The concentration of erythrocytes and hemoglobin content increase in individuals habitually occupying the plateau (a hypoxic region) due to the acclimatization and CMS, and there is a positive correlation between the amount of hemoglobin and cerebral vessel density. Therefore, it is a physiological phenomenon (i.e. acclimatization to high altitude) that the residents living in the plateau have a higher density of blood vessels and cranial nuclei than the ones living in the plains. As such, this phenomenon should be distinguished from the residue of the contrast agent or pathological conditions such as subarachnoid hemorrhage, hyperdense artery sign, and venous sinus thrombosis, in order to prevent excessive diagnosis.

## Abbreviations

CT: Computed tomography
MCH: Mean corpuscular hemoglobin
MCHC: Mean corpuscular hemoglobin concentration
CMS: Chronic mountain sickness
